# Cholesterol-like effects of a fluorotelomer alcohol incorporated in phospholipid membranes

**DOI:** 10.1038/s41598-018-20511-0

**Published:** 2018-02-01

**Authors:** Mark Jbeily, Ruth Bärenwald, Jörg Kressler, Kay Saalwächter, Tiago Mendes Ferreira

**Affiliations:** 1 Institute of Physical Chemistry, Martin-Luther-University Halle-Wittenberg, 06120 Halle, Germany; 2 Institute of Physics, Martin-Luther-University Halle-Wittenberg, 06120 Halle, Germany

## Abstract

Fluorocarbon amphiphiles are anthropogenic substances widely used in diverse applications such as food packaging, clothing or cookware. Due to their widespread use and non-biodegradability, these chemicals are now ubiquitous in the natural world with high propensity to bioaccumulate in biological membranes, wherein they may affect microscopic properties. Here, we test the hypothesis that a typical fluorocarbon amphiphile can affect lipid membranes similarly to cholesterol by investigating the effect of 1*H*,1*H*,2*H*,2*H*-perfluoro-1-decanol (8:2 FTOH) on 1,2-dipalmitoyl-*sn*-glycero-3-phosphocholine (DPPC) membranes. Using solid-state nuclear magnetic resonance spectroscopy, differential scanning calorimetry and confocal microscopy, we present a consistent set of independent experimental evidences supporting this hypothesis, namely that upon incorporation of 8:2 FTOH, (i) a condensing effect on the acyl chains occurs in the fluid phase, (ii) coexistence of two membrane phases is observed below melting, and (iii) the melting temperature of DPPC varies no more than approximately ±1 °C up to a concentration of 40 mol% of 8:2 FTOH. The condensing effect is quantified by means of advanced dipolar recoupling solid-state NMR experiments and is found to be of approximately half the magnitude of the cholesterol effect at the same concentration.

## Introduction

Fluorocarbons are anthropogenic substances that can combine a number of interesting properties such as high surface activity, simultaneous lipophobic and hydrophobic behavior, biochemical inertness, chemical stability and high oxygen solubility^1,2^. Such unique combination of properties led, for instance, to the design of perfluorocarbon-based surfaces for diverse applications (e.g. TEFLON for cookware or GORE-TEX for clothing), to the development of fluorocarbon-based nanoparticles to use in photodynamic therapy^3^, to fluorocarbon-based liquid ventilation methods^4^ and to synthetic blood^5,6^.

Due to the widespread use of fluorocarbons in the last few decades, especially of amphiphiles with perfluorocarbon moieties, these compounds are now regularly found in the blood of animals and humans^7^. Perfluorooctanesulfonic acid (PFOS) is the most abundant fluorocarbon in the natural world and has been detected in various wildlife aquatic mammals, with the highest levels (≈1.3 *μ*g/g) found in the liver of polar bears^8^. PFOS production was discontinued by the main producer in 2000 because of its potential bioaccumulation and consequent health risks^7^. Other amphiphilic fluorocarbons, such as perfluorooctanoic acid (PFOA) or fluorotelomer alcohols (FTOHs), are still produced in large scale for a number of applications and are being consequently released in the environment^9,10^. For instance, in Japan, FTOHs were detected with concentrations of a few ng/m^3^ in indoor air^11^, the most abundant being 1*H*,1*H*,2*H*,2*H*-perfluoro-1-decanol (8:2 FTOH). Due to the increase of amphiphilic fluorocarbons in the environment, there is a growing concern about their toxicology^12,13^. At present, however, the long term risk on human health from the current exposure to these molecules is mainly unknown^14–16^.

There is mounting evidence that amphiphilic molecules containing perfluorocarbon moieties, such as perfluorooctanesulfonic acid (PFOS), perfluorooctanoic acid (PFOA) or fluorotelomer alcohols, will incorporate in cell membranes due to the hydrophobicity of their fluorinated chains. However, the reported effects vary, and are even partially contradictory. While PFOS has been reported to incorporate into mitochondrial membranes and increase their fluidity^17^, PFOA was found to induce a slight ordering in 1,2-dimyristoyl-*sn*-glycero-3-phosphocholine (DMPC) bilayers, implying a weak stiffening^18^. In contrast, dynamic light scattering and neutron-spin echo experiments on PFOA in DMPC bilayers were interpreted in terms of a pronounced stiffening, originating from a condensing effect reminiscent of the mode of action of cholesterol^19^. However, such discrepancies could arise from distinct sample preparations, which could affect the level of protonation/deprotonation of PFOA and consequently the location of PFOA within the bilayer (see e.g. ref.^20^). It was also suggested that highly fluorinated alcohols such as CF_3_(CF_2_)_7_(CH_2_)_5_ OH (8:5 FTOH) may have similar properties to cholesterol in 1,2-dipalmitoyl-*sn*-glycero-3-phosphocholine (DPPC) monolayers^21^.

Contrarily to the highly dynamic nature of fatty acids and *n*-alcohols, PFOA and fluorotelomer alcohols have stiff and helical perfluorinated chains^22^. When incorporated in a membrane, these amphiphilic fluorocarbons may therefore behave essentially as amphiphilic rigid rods anchored to the lipid-water interface thereby inducing order on the neighbouring phospholipid acyl chains. In the context of lung surfactant replacement therapies, for which compounds with fluorinated chains have been tested due to their high oxygen solubility, the analogy with cholesterol is highly important since it has recently been shown that the incorporation of cholesterol in lung surfactant extracts severely affects its phase behaviour^23^. Moreover, such an analogy may be important to understand the biological effects of fluorocarbon amphiphiles on cellular membranes, where cholesterol plays an essential role.

**Figure 1:**
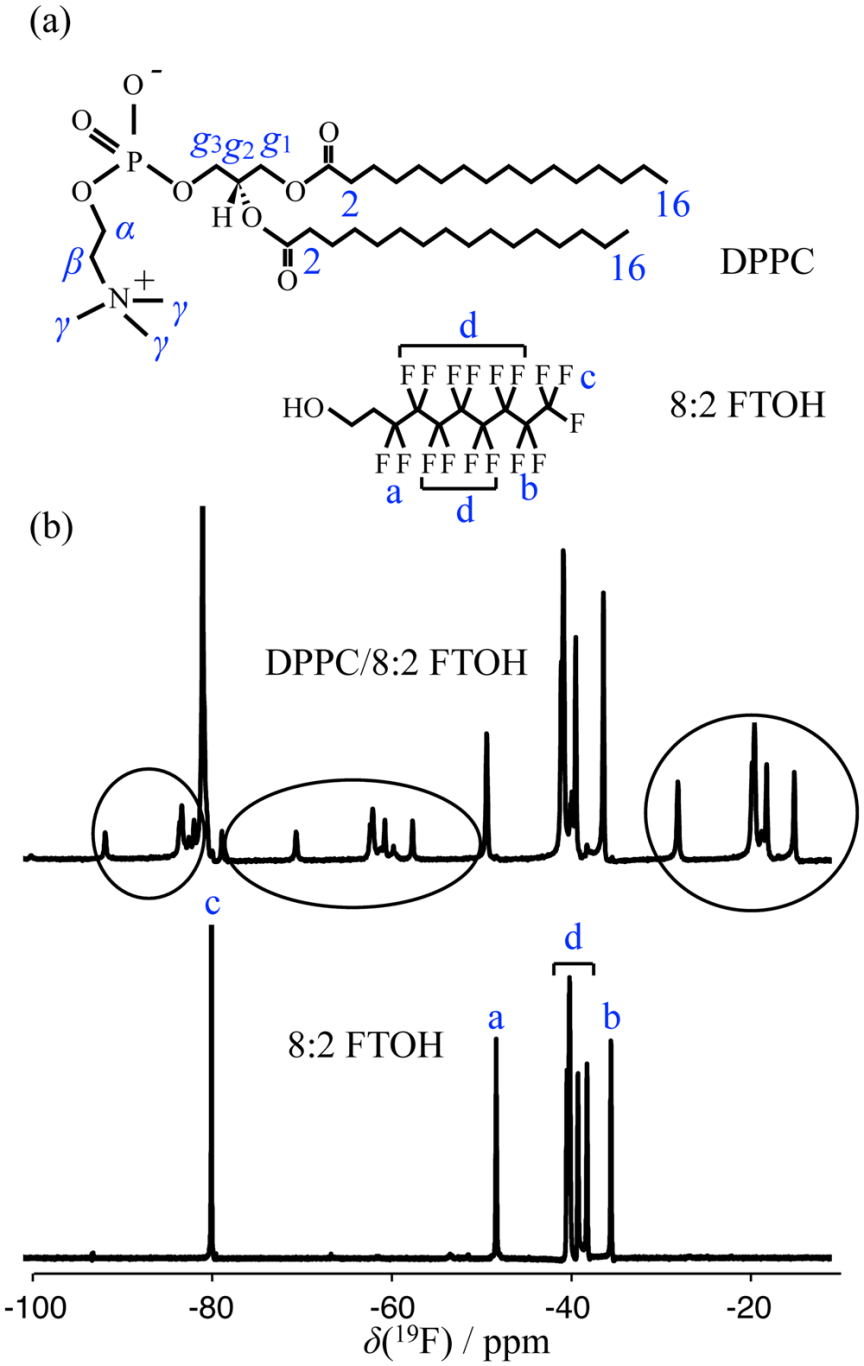
(**a**) Molecular structures of DPPC and 8:2 FTOH, and labels used for referring to the individual carbons of DPPC and to the fluorinated carbon segments in 8:2 FTOH. (**b**) ^19^F CPMG-filtered solid-state NMR spectra of DPPC/8:2 FTOH (60:40 mol %) MLVs (top) and neat 8:2 FTOH (bottom) at a temperature of 60 °C. Spinning sidebands are depicted inside ellipses. The CPMG filter was used to remove the ^19^F signal from the MAS rotor inserts made of KEL-F.

In this work, we investigate such a possible condensing effect by fluorinated rigid rods in lipid membranes, namely of 8:2 FTOH in DPPC lipid bilayers (molecular structures in Fig. 1a), using solid-state nuclear magnetic resonance (NMR) spectroscopy, differential scanning calorimetry (DSC) and confocal laser scanning microscopy (CLSM). DPPC is the major component of lung surfactant and DPPC/cholesterol is one of the most studied lipid membrane systems until date. The atomistic-level details from the solid-state NMR experiments enable to unequivocally demonstrate that the fluorinated rigid rods incorporate in the DPPC bilayer inducing a significant ordering of the acyl chains. Moreover, the complete set of results presented indicates that the effect of 8:2 FTOH on the DPPC molecular structure is reminiscent of the effect of cholesterol and other sterols, both below and above the DPPC melting temperature. This suggests that the temperature-composition phase diagram of the DPPC/8:2 FTOH (DPPC/rigid-rod) system and of such DPPC-sterol systems might be similar.

## Results and Discussion

A clear evidence that 8:2 FTOH molecules can incorporate in DPPC lipid bilayers is the observation of anisotropic features in the ^19^F NMR spectra of the DPPC/8:2 FTOH multilamellar vesicles (MLVs) under magic-angle spinning (MAS) as shown in Fig. 1b. The appearance of spinning sidebands in the ^19^F spectrum demonstrates the anisotropic motion of 8:2 FTOH in contrast to its apparent isotropic motion in a neat sample above its melting temperature.

**Figure 2:**
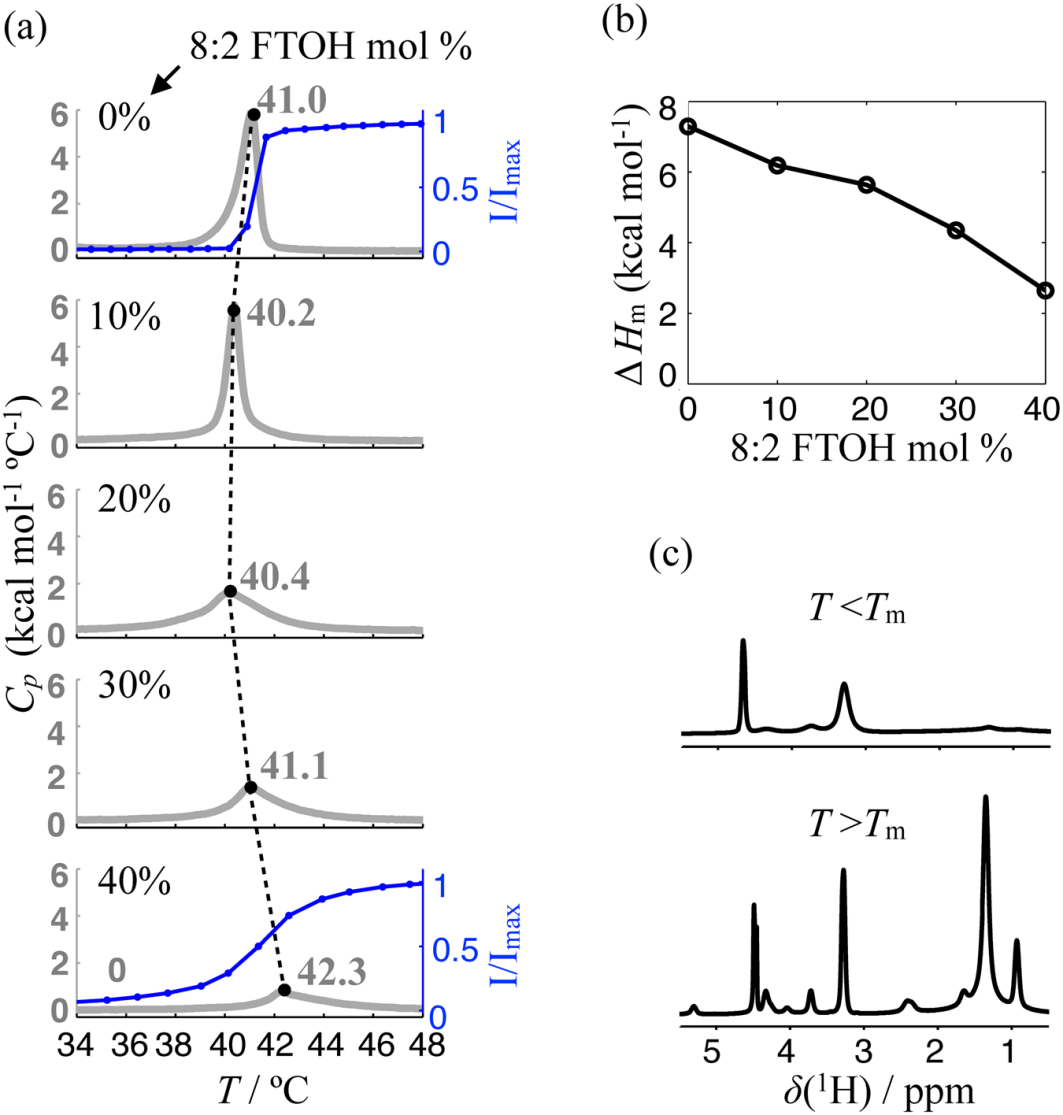
(**a**) DSC heating curves (1 °C/min) for a number of DPPC/8:2 FTOH mixtures with different amounts of fluorotelomer alcohol plotted together with corresponding profiles for the maximum intensity in the interval 1–2 ppm of the ^1^H NMR spectrum. The variation of the melting temperatures, *T*_*m*_, in the DSC traces is depicted as a dashed line with values indicated. (**b**) The enthalpy of transition, Δ*H*_m_, calculated from the DSC traces and normalized by total mole number of lipid and 8:2 FTOH in the corresponding samples. (**c**) Examples of solid-state ^1^ H NMR spectra of pure DPPC MLVs in the gel phase (top) and the fluid lamellar phase (bottom).

The measured DSC profiles from a number of DPPC/8:2 FTOH mixtures are shown in Fig. 2a. All DSC traces display a single melting temperature (*T*_*m*_) indicating complete incorporation up to 40 mol % of 8:2 FTOH in the lipid MLVs, i.e. showing no occurrence of macroscopic phase separation in the samples since otherwise more than a single DSC peak would be expected. Here, we always refer to *T*_*m*_ as the position of the maximum in the DSC traces. At a concentration of 10 mol % 8:2 FTOH there is a slight downshift of *T*_*m*_ and, as for the case of DPPC MLVs, the transition is of first order. With increasing 8:2 FTOH concentration the melting temperature then gradually upshifts and the transition becomes rather gradual over broad temperature intervals. The melting temperature shifts no more than approximately ±1 °C throughout the entire concentration interval.

The DSC features observed are reminiscent of the well known DSC profiles of DPPC MLVs with sterols^24–29^. Namely, for low concentrations of sterol a slight downshift of the melting temperature is observed which levels off at about 7.5 to 20 mol %; with further increasing concentration of sterol, the DSC peak broadens and slightly shifts to higher temperatures. We shall therefore explore further the hypothesis that an amphiphilic rigid-rod, such as 8:2 FTOH, and sterols, have similar effects on DPPC membranes and compare our results with results previously obtained for DPPC/sterol systems.

According to the interpretation of DSC and ^2^H NMR results by Vist and Davis^25^ and to the thermodynamic and microscopic interaction models by Ipsen *et al*.^24^, the DPPC/cholesterol phase diagram contains a eutectic point which connects three regions of two-phase coexistence (see Supplementary Fig. 1). The phase diagrams of DPPC with other sterols that have been reported are very similar with slight variations of the borders that define the two-phase regions^28,29^. One apparent contrast between the DPPC/sterol and DPPC/8:2 FTOH systems is that for the former, above the concentration of sterol which defines the rich-sterol boundary of the *S*_o_ + *L*_o_ region (about 20 to 30 mol % in the membrane^24,25,28,29^), the gel to liquid crystalline phase transition is abolished. On the other hand, our DSC trace for the DPPC bilayers with 40 mol % of 8:2 FTOH clearly displays an apparent phase transition. Such phase transition is supported by our ^1^H solid-state NMR results shown together with the DSC traces in Fig. 2a, where the normalized intensity of the main methylene peak, *I*/*I*_max_, in the ^1^H spectra is plotted against temperature for DPPC and DPPC/8:2 FTOH (60:40 mol %) MLVs. The increase in *I*/*I*_max_ is due to the transition from the slow dynamics of the acyl chains in the gel phase to the faster axially symmetric motions in the liquid crystalline phase and is observed for both the pure DPPC MLVs and for the DPPC/8:2 FTOH (60:40 mol %) MLVs. Note, however, that such an apparent phase transition is also present in the DSC traces of DPPC/cholesterol MLVs (60:40 mol %) reported by Huang *et al*.^27^ and by McMullen and McElhaney^26^, although with a much lower cooperativity (i.e. with a larger temperature interval for the phase transition) in comparison to the DPPC/FTOH case. In fact, Huang *et al*.^27^ and McMullen and McElhaney^26^ report DPPC/cholesterol phase diagrams with phase transitions at a concentration of 40 mol % of cholesterol (contrarily to the phase diagram by Ipsen *et al*.^24,25^ and Vist and Davis^24,25^).

The transition enthalpy, Δ*H*_m_, of a DPPC/cholesterol mixture at 60:40 mol % is between 2.5–4 kcal/mol^27^ which is surprisingly similar to the value measured for the DPPC/8:2 FTOH MLVs at 60:40 mol % as shown in Fig. 2b. This would indicate that the enthalpy is mostly dominated by DPPC-DPPC interactions disturbed similarly by cholesterol and 8:2 FTOH, and/or that the DPPC-cholesterol and DPPC-8:2 FTOH interactions are of similar magnitude. It is not clear though if the value reported by Huang *et al*.^27^ is normalized by number of DPPC molecules or by total number of DPPC and cholesterol molecules. If the former case was used, then the enthalpy of transition for the DPPC/8:2 FTOH mixture is higher.

The main difference that we observe in the DSC traces between the DPPC/FTOH and the previously published results for DPPC/cholesterol is the temperature width of the transition. For the DPPC/sterol (60:40 mol %) mixtures such temperature width is equal to about 60 °C for the DPPC/cholesterol case^27^, while it is about 10 °C for DPPC/8:2 FTOH (60:40 mol %) as shown in Fig. 2a. Interestingly, it has been shown that the temperature width of the DSC peak for phosphatidylcholine/cholesterol systems at 60:40 mol % decreases by increasing the length of the phospholipid acyl chains^27^, i.e. by increasing the free volume in the bilayer core the cooperativity increases. This could be due to the occurence of interdigitation between the acyl chains of opposite bilayer leaflets, which could stabilize the gel phase, and/or to an increase of disorder in the liquid phase induced by the increase of free volume. Replacing cholesterol by 8:2 FTOH in a DPPC bilayer also induces an increase of free volume in the bilayer core since the fluorotelomer is shorter than cholesterol inducing similarly a narrowing of the DSC peak in comparison to the DPPC/cholesterol case.

To find evidence for the coexistence of two distinct lipid phases below *T*_*m*_ at a certain interval of fluorotelomer fraction in the membranes, as expected from the DPPC/sterol phase diagram (Supplementary Fig. 1), we performed CLSM experiments at room temperature of DPPC/8:2 FTOH giant unilamellar vesicles (GUVs) double stained with ATTO-633-DOPE, a commonly used red-dye which partitions preferentially in disordered lipid environments, and Rh-C_2_ H_4_-C_10_ F_21_, a synthesized rhodamine based fluorinated fluorescence dye^30^ that has been shown to partition preferentially in fluorine rich phases^31^. As it can be seen in Fig. 2(a) and already described above, the ^1^H NMR relaxation is very fast below melting temperature which makes the use of NMR for investigating gel phases difficult. The CLSM experiments on the other hand enable a clear picture of phase coexistence if the probes used have preference for one the coexisting phases.

**Figure 3:**
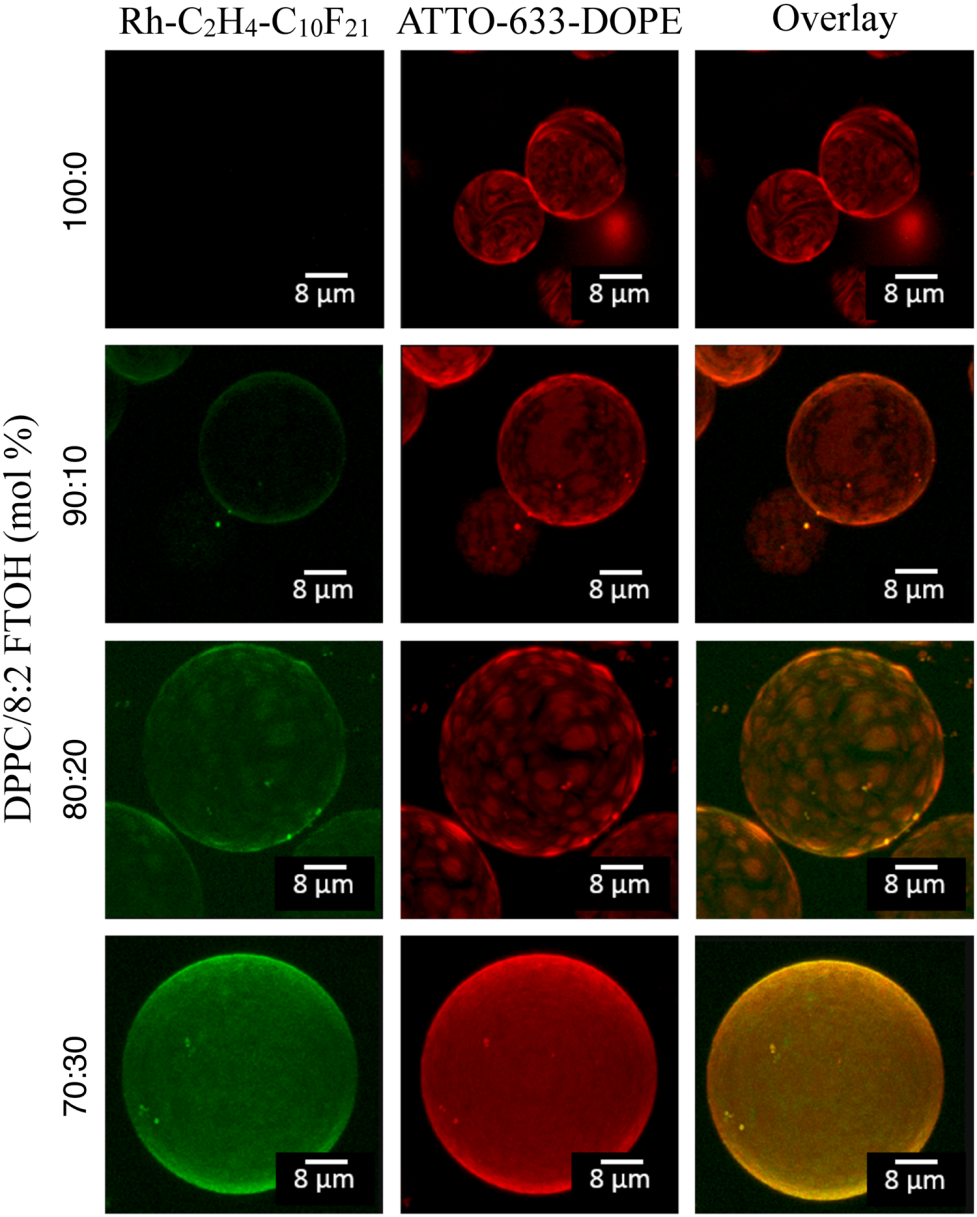
CLSM z-stacking images of DPPC and DPPC/8:2 FTOH mixed GUVs using double staining with a synthethised rhodamine based fluorinated fluorescence dye (0.5 mol % Rh-C_2_ H_4_-C_10_ F_21_, green channel, left) that partitions preferentially in phases containing fluorinated molecules^30,31^ and a commonly used dye (0.5 mol % ATTO-633-DOPE, red channel, middle) used to stain disordered phases. The images on the right are overlays of both channels.

As can be seen in Fig. 3, pure DPPC GUVs are faceted at room temperature, with facets corresponding to highly ordered gel phase regions connected by fringes where the DPPC acyl chains are less ordered and where the ATTO-633-DOPE preferentially locates^32^. The fluorinated-dye is only detected from the GUVs with 8:2 FTOH. Phase coexistence is clearly observed for the GUVs with 20 mol % of 8:2 FTOH for which the CLSM pictures show the presence of *μ*m size domains located in a continuous phase, both through the ATTO-633-DOPE channel (red, middle) and the Rh-C_2_ H_4_-C_10_ F_21_ channel (green, left). The overlay of the pictures from the different channels is also provided (right) showing a preferred partitioning of the Rh-C_2_ H_4_-C_10_ F_21_-dye in the ATTO-633-DOPE-rich regions. This indicates that, at room temperature, the 8:2 FTOH-rich phase is more disordered than a pure DPPC phase.

**Figure 4:**
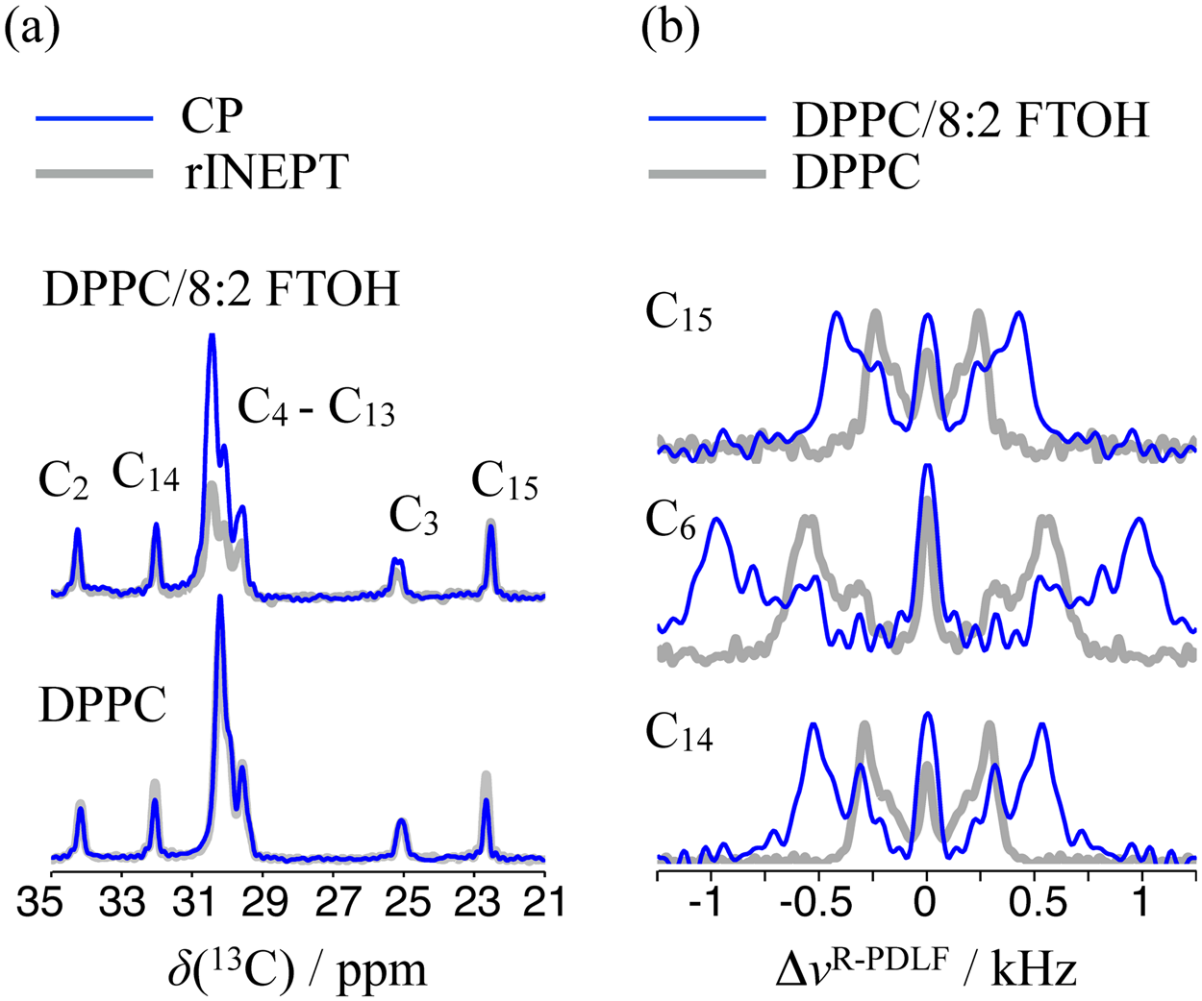
(**a**) rINEPT (grey) and CP (blue) spectra of DPPC/8:2 FTOH (60:40 mol %) and DPPC MLVs above *T*_*m*_. The labels used to identify the DPPC peaks refer to Fig. 1a. (**b**) Selected dipolar cross sections from the ^1^H–^13^C R-PDLF spectra of DPPC/8:2 FTOH (60:40 mol %) (blue) and DPPC (grey) multilamellar vesicles.

If the effect of the fluorotelomer alcohol on the DPPC phase structure is similar to the efect of sterols, then the perfluoroalkyl rigid-rod should have a condensing effect on the acyl chains in the liquid crystalline phase, i.e. the presence of 8:2 FTOH in the bilayer should increase the population of DPPC acyl chain conformations with higher content of trans states consequently increasing the thickness of the fluid membrane (in case inter-digitation does not occur). The effect of 8:2 FTOH on the structure of the DPPC acyl chains can be investigated qualitatively by comparing the spectra of pure DPPC and DPPC/8:2 FTOH MLVs acquired using refocused insensitive nuclei enhanced polarisation transfer (rINEPT) and cross polarisation (CP) as shown in Fig. 4a. For pure DPPC MLVs, CP gives always the same intensities or less than rINEPT. On the other hand, for the DPPC/8:2 FTOH system the CP transfer offers higher signal enhancement for many acyl chain segments. This result shows qualitatively that the inclusion of 8:2 FTOH in the membrane orders the DPPC acyl chains since an ordering of the acyl chains increases the CP and decreases the rINEPT efficiencies. For a theoretical description of the dependency of CP and rINEPT intensities on acyl chain ordering see e.g. Nowacka *et al*.^33^.

**Figure 5:**
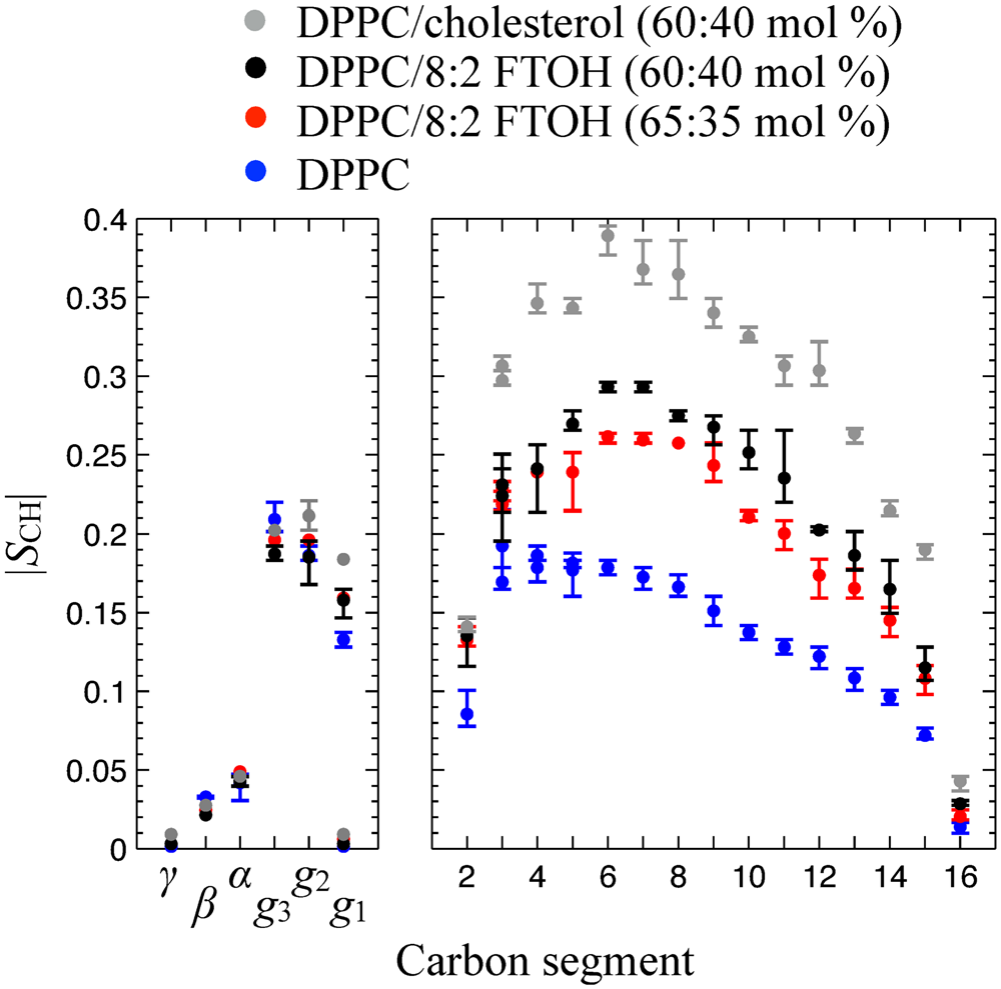
Carbon segmental profiles of order parameter magnitudes, |*S*_CH_|, determined with R-PDLF spectroscopy for a pure DPPC bilayer (blue) and for DPPC bilayers with 35 mol % (red) and 40 mol % (black) of 8:2 FTOH, and 40 mol % of cholesterol (grey). The points and error bars plotted are defined as described schematically in the Fig. S4 given as supplementary information.

To quantify the ordering effect, we use R-PDLF NMR spectroscopy^34–36^ which enables to measure the ^1^H–^13^C dipolar splittings of each C–H bond in DPPC and consequently determine how the inclusion of the perfluorinated chains affects the C–H bond order parameter magnitudes,

1 $$|{S}_{{\rm{CH}}}|=|1/2\langle 3\,{\cos }^{2}\theta -1\rangle |,$$

where *θ* is the angle between the C–H bond and the bilayer normal (|*S*_CH_| can be calculated from a R-PDLF dipolar splitting as described in the Materials and Methods section). Figure 4b shows three selected slices of the R-PDLF spectra of DPPC and DPPC/8:2 FTOH MLVs and Fig. 5 shows the C-H bond order parameter profiles determined for a number of samples (the full set of R-PDLF spectra and dipolar slices from which the order parameter profiles were derived is shown in the Supplementary Figs 3–7). The strategy for assigning order parameters to the many carbons in the crowded spectral region at 29–31 ppm is described in the Materials and Methods section. The presence of the alcohol induces a significant increase to the |*S*_CH_| profile of the DPPC acyl chains while the DPPC headgroup |*S*_CH_| values remain constant. Again, the effect is reminiscent to the well known condensing effect of cholesterol^25^, although the ordering effect is about half the magnitude of the cholesterol effect with respect to |*S*_CH_| magnitudes as shown in Fig. 5. Also similarly to the effect of cholesterol^36,37^, the headgroup order parameters are not affected by the inclusion of FTOH which suggests that the hydroxyl group is located near the acyl chain oxygens and the fluorinated part of the molecule is oriented along the acyl chains.

There have been previous suggestions that molecules with perfluoroalkyl rigid-rods as in 8:2 FTOH may have similar effects to cholesterol in lipid bilayers and monolayers^19,21^. As many of the amphiphilic molecules with perfluorocarbon moieties present in the environment, the 8:2 FTOH studied has a rigid linear chain of eight perfluorinated carbons in contrast to the cyclic sterols. Our experimental results suggest therefore that the known condensing effect by cholesterol on lipid bilayers is not exclusive for sterols and/or specific phospholipid-sterol interactions, but most likely extends also to simple amphiphilic rigid rods such as amphiphiles with perfluorocarbon moieties such as 8:2 FTOH. The low polarizability of C-F bonds, together with the chain rigidity due to the high energetic cost for dihedral rotations around -CF_2_-CF_2_-CF_2_- bonds, makes perfluoroalkyl chains to have very weak intermolecular interactions with hydrocarbons, which is the basis for its lipophobicity. Therefore, the results presented here strongly indicate that simple steric interactions of highly fluorinated rigid rods inserted in a lipid bilayer are sufficient to induce a significant ordering effect on the phospholipid acyl chains.

The present use of fluorocarbon amphiphiles for a number of applications causes the accumulation of such molecules in the environment and in living organisms. The levels of fluorocarbon amphiphiles in human serum have been reported in the year 2008 to be around 10 to 70 ng/ml with substantial variations throughout different regions^7^. In comparison to the cholesterol levels found in human serum, the estimate is then that the number of fluorocarbon amphiphiles in human serum is on the order of 0.001% of the total number of cholesterol molecules. The distribution of fluorocarbon amphiphiles over different cellular membranes and tissues is however presently unknown. Cholesterol homeostasis is highly important because the amount of cholesterol in cellular membranes substantially affects membrane properties such as lateral lipid heterogeneity, lipid diffusion and membrane thickness to mention a few. The accumulation of fluorocarbon amphiphiles in cellular membranes will therefore also affect such properties. At present it cannot be judged if these effects are significant with the present concentrations of fluorocarbon amphiphiles found in living organisms. However, an increase of the amount of such molecules in the environment and subsequent bioaccumulation in living organisms constitutes a risk to health concerning the variation of biomembrane properties that may be foreseen with basis on the physical-chemistry study here presented.

## Materials and Methods

### Sample preparation

The preparation of samples was done with the following chemicals as described below. The phospholipid 1,2-dipalmitoyl-*sn*-glycero-3-phosphocholine (DPPC) was purchased from Avanti Polar Lipids, the fluorotelomer alcohol 1H,1H,2H,2H-perfluoro-1-decanol (8:2 FTOH), deuterated chloroform (CDCl3) and D_2_ O from Sigma-Aldrich, non-deuterated chloroform from Carl Roth, and the fluorescence dye ATTO-633-DOPE from ATTO-TEC GmbH. The rhodamine based fluorinated fluorescence dye Rh-C_2_ H_4_-C_10_ F_21_ was synthesized according to a previously reported procedure^30^.

The DPPC/8:2 FTOH lipid films for the DSC and NMR experiments were prepared by mixing chloroform solutions of DPPC and 8:2 FTOH in the right amounts and then rapidly evaporating the solvent in a rotary evaporator. The lipid film was afterwards further dried in a vacuum oven overnight. For the DSC measurements the lipid films were then ultrasonicated in deionized water (0.055 *μ*S/cm) to a final lipid concentration of 1.09 mM. For the NMR measurements the lipid films were hydrated with 40 %wt in total of deuterated water in 0.5 mL EPPENDORF tubes performing cycles of heating, stirring and centrifuging repeatedly until homogenous samples were obtained which were then centrifuged to 4 mm solid-state NMR rotors. A small fraction of the lipid films used for preparing the NMR samples was dissolved in deuterated chloroform and analysed with high resolution (HR) NMR in order to determine the final DPPC/8:2 FTOH ratio in the lipid films. Because of the high volatility of 8:2 FTOH, the lipid films had a lower fraction of 8:2 FTOH than the initial ratio before solvent evaporation.

GUVs were prepared by electroformation similarly to previously published protocols^38^. 10 *μ*L of DPPC or DPPC/8:2 FTOH chloroform solutions having a concentration of 10 mg/mL lipid was spin coated on an ITO coated glass disk purchased from Präzisions Glas & Optik GmbH, Germany (25.2 mm diameter and 0.175 mm thickness) at 2000 rpm for 1 min at room temperature. Conducting copper tape (3 M single-sided adhesive, 1/4 inch width) was used to connect the ITO-covered glass disk to the low-frequency generator (LFG). Silicon grease was used to seal the flow chamber and make it water-tight. ROTILABO-PE tube with inner diameter of 1 mm and outer diameter of 1.8 mm, purchased from Carl Roth GmbH, was used to fill the flow cell with degased deionized water. The flow cell was heated on a heating plate to 62 °C above the main transition temperature of DPPC^39^ and electroformation was performed with a sinusoidal alternating current having an effective voltage of 1.3 V and a frequency of 10 Hz supplied by the LFG for 3 h. After electroformation, the flow cell was removed from the heating plate and left to cool down to room temperature before examination with the the confocal microscope.

### Differential Scanning Calorimetry

Differential Scanning Calorimetry (DSC) thermograms were measured with a Microcal VP-DSC (MicroCal Inc., Northhampton, USA) and the reference cell was filled with deionized water. Heating rates were 1 °C/min and the second heating cycle was used which was always identical to the third heating cycle. Data processing was done with MATLAB by performing baseline corrections with a polynomial of degree 5 after subtraction of the reference cell DSC trace. The melting enthalpies were calculated from the area under the endotherm.

### Confocal Laser Scanning Microscopy

A Leica TCS SP2 DM IRE2 confocal microscope equipped with an HCX PL APO lbd.BL 63 × 1.2 W CORR (water immersion) objective (Leica Microsystems, Wetzlar, Germany) was used. The ATTO-633-DOPE probe (0.5 mol %) was used to stain the GUVs and excited with a 633 nm HeNe laser and detected via the 550–695 nm red channel detection window. The rhodamine based fluorinated fluorescence dye probe Rh-C_2_ H_4_-C_10_ F_21_ (0.5 mol %) was excited with the 488 nm Ar-Kr laser and detected via the 510–550 nm green channel detection window. Imaging of the GUVs was performed at room temperature and z-stacking was done from bottom to top. Images were processed with the Leica software. The DPPC/8:2 FTOH 60:40 mol % mixture did not yield GUVs most likely due to the effect of the fluorotelomer on the curvature properties of the bilayers, namely the increase of stiffness of the bilayer induced by the presence of the FTOH. We note also that the collection of pictures acquired showed consistently that mixed DPPC/8:2 FTOH systems yield more GUVs than with pure DPPC.

### Solid-state Nuclear Magnetic Resonance

All experiments were performed using a Bruker Avance III 400 spectrometer operating at a ^1^H Larmor frequency of 400.03 MHz equipped with a standard 4 mm CP-MAS HX probe. The temperatures of all the solid-state NMR experiments performed were calibrated with a methanol sample spinning at the MAS frequencies used^40^.

The ^19^F Carr-Purcell-Meiboom-Gill (CPMG)^41,42^ experiments were acquired under a magic angle spinning (MAS) frequency of 8 kHz, using a total number of six 180° pulses with nutation frequency of 78.12 kHz, delays between pulses equal to the MAS time period, a recycle time of 5 s, acquisition time of 0.1 s and a total number of 64 scans.

The ^1^H direct polarization experiments were done using a nutation frequency of 78.12 kHz, a recycle time of 5 s, acquisition time of 0.5 s, and a total number of 4 scans.

#### *R-PDLF experiments to measure* |*S*_CH_| *values*

The result of a ^1^H–^13^C R-PDLF experiment is a 2D spectrum with carbon chemical shift resolution in the direct dimension and recoupled dipolar line shapes in the indirect dimension featuring a dipolar splitting Δ*ν*^R−PDLF^. The ^13^C chemical shift-resolved dipolar splittings in the 2D spectrum are proportional to the magnitudes of order parameters *S*_CH_ of their corresponding C–H bonds by

2 $${\rm{\Delta }}{\nu }^{{\rm{R}}-{\rm{PDLF}}}=0.315{d}_{{\rm{CH}}}^{{\rm{\max }}}|{S}_{{\rm{CH}}}|$$

with 0.315 as a scaling factor specific of the dipolar recoupling sequence used and ${d}_{{\rm{CH}}}^{{\rm{\max }}}$ the rigid dipolar coupling constant equal to 21.5 kHz^34^. Note that there are cases for which the C–H bonds of a methylene segment are inequivalent and that in such cases the two distinct C–H bond order parameters can be determined with R-PDLF spectroscopy. This methodological approach is superior to the conventional use of ^2^H NMR on perdeuterated samples because it enables to separate ^1^H–^13^C heteronuclear dipolar spectra from the carbon chemical shifts in two dimensions which results in a higher resolution of the distinct acyl chain carbons (see e.g. Fig. S3 and references^20,23,36,43–49^). The carbons of the crowded spectral region at 29–31 ppm were assigned having the previous assignment for 1-palmitoyl-2-oleoyl-sn-glycero-3-phosphocholine (POPC) by Ferreira *et al*. as reference^36^. The previous assignment by Ferreira *et al*.^36^ was done by using two distinct samples, one with POPC molecules having natural abundance of isotopes and another with the *sn*-1 chain of POPC fully deuterated in combination with molecular dynamics simulation. There is a high similarity of the 2D R-PDLF spectrum for the crowded spectral region of POPC (Fig. 2 in reference^36^) and DPPC (Supplementary Fig. 3). Note that in reference^36^ the carbon chemical shift scale in ppm was calibrated by defining the acyl chain methyl peaks at 13.9 ppm while the chemical shift scale shown in this work was calibrated using an alanine sample which gave a value of 14.3 ppm for the DPPC acyl chain methyl peaks. Taking this into account, one observes that the splittings in the crowded spectral region of POPC and DPPC MLVs at 27 °C and 52 °C, respectively, have very similar chemical shifts. Although our assignment is not foolproof it is the most reasonable choice to convey the data according to the information presently known and more informative than simply taking the assumption of monotonic decrease of order parameters along the acyl chain, which is commonly done in ^2^NMR studies using perdeuterated chains. The full assignment of the splittings in the R-PDLF spectrum of all the studied samples (including the splittings in the crowded spectral region) is shown as Supplementary Information. Moreover, Fig. S4 shows schematically how we calculated the order parameters from the spectrum, as well as how the error bars in Fig. 5 are defined.

We define the pulse sequence parameters according to Figs 1c and 2c of the original reference for the R-PDLF experiment^34^. The MAS frequency used was 5.15 kHz and *t*_1_ increments used were 388.35 *μ*s equal to twice the MAS rotor period for all samples except for the DPPC/cholesterol MLVs for which *t*_1_ increments equal to 258.9 *μ*s were used. The refocused-INEPT^50,51^ delays *τ*_1_ and *τ*_2_ were multiples of the MAS rotation period, namely 1.94 ms and 0.97 ms, respectively. The RF pulses used had the following nutation frequencies: 46.35 kHz (${\rm{R}}{18}_{1}^{7}$ pulses), 63.45 kHz (^13^C 90° and 180° pulses) and 50 kHz (SPINAL64 ^1^H decoupling^52^). A long recycle delay of 8 s was used to prevent RF heating. The total acquisition time for each scan in the direct dimension was 0.1 s using a spectral width of 200 ppm. The total number of points in the indirect dimension and total number of scans used for each sample are given in the Supplementary Figs S3–S6 below.

## Electronic supplementary material

Supplementary Information
