# Supplementary material for: Cholesterol-like effects of a fluorotelomer alcohol incorporated in phospholipid membranes
Source: Sci Rep. 2018 Feb 1;8:2154. doi: 10.1038/s41598-018-20511-0 (PMC5794869; doi:10.1038/s41598-018-20511-0)
Supplement: Supplementary file 1 — Supplementary Information [file 41598_2018_20511_MOESM1_ESM.pdf]

Supplementary Information:
Cholesterol-like effects of a fluorotelomer alcohol incorporated in phospholipid membranes

M. Jbeily,[1] R. Bärenwald,[2] J. Kressler,[1] K. Saalwächter,[2] and T. M. Ferreira[2,*]

[1]*Institute of Physical Chemistry, Martin-Luther-Universität Halle-Wittenberg, Germany*
[2]*Institut für Physik - NMR, Martin-Luther-Universität Halle-Wittenberg, Germany*

(Dated: November 8, 2017)

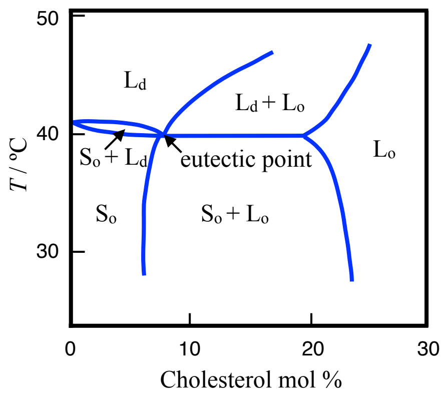

FIG. 1: Temperature-composition diagram of the DPPC/cholesterol system at full hydration adapted from Ipsen et al (reference 25 in the main paper). The labels L_d and L_o, are used to denote the disordered and ordered liquid crystalline lamellar phases, respectively, while S_o denotes a lamellar phase with a solid-like nature.

* tiago.ferreira@physik.uni-halle.de

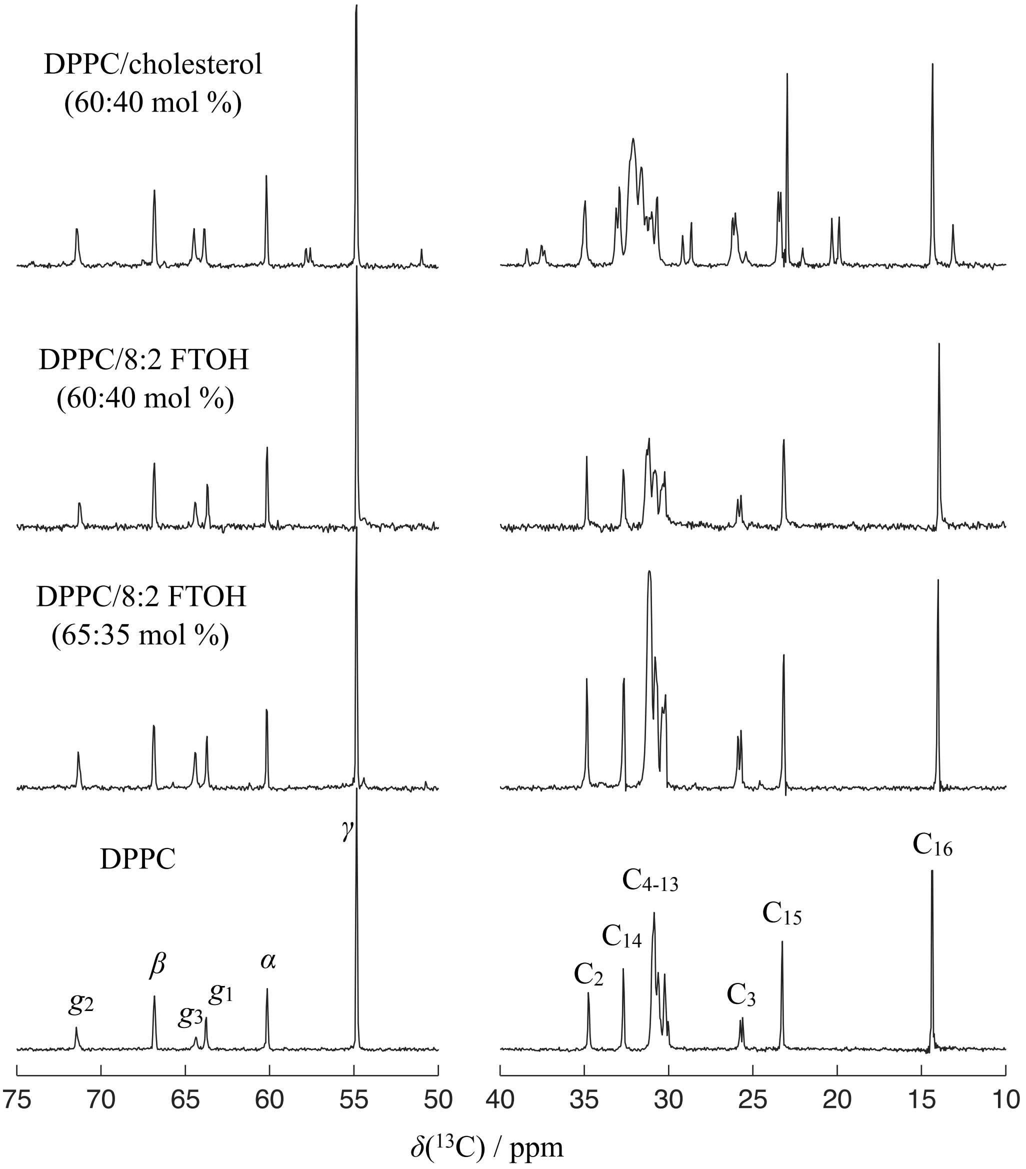

FIG. 2: Refocused INEPT spectra from the different samples studied acquired at a temperature of 52 °C. All the spectra are normalised such that the maximum intensity of the γ peak is unity.

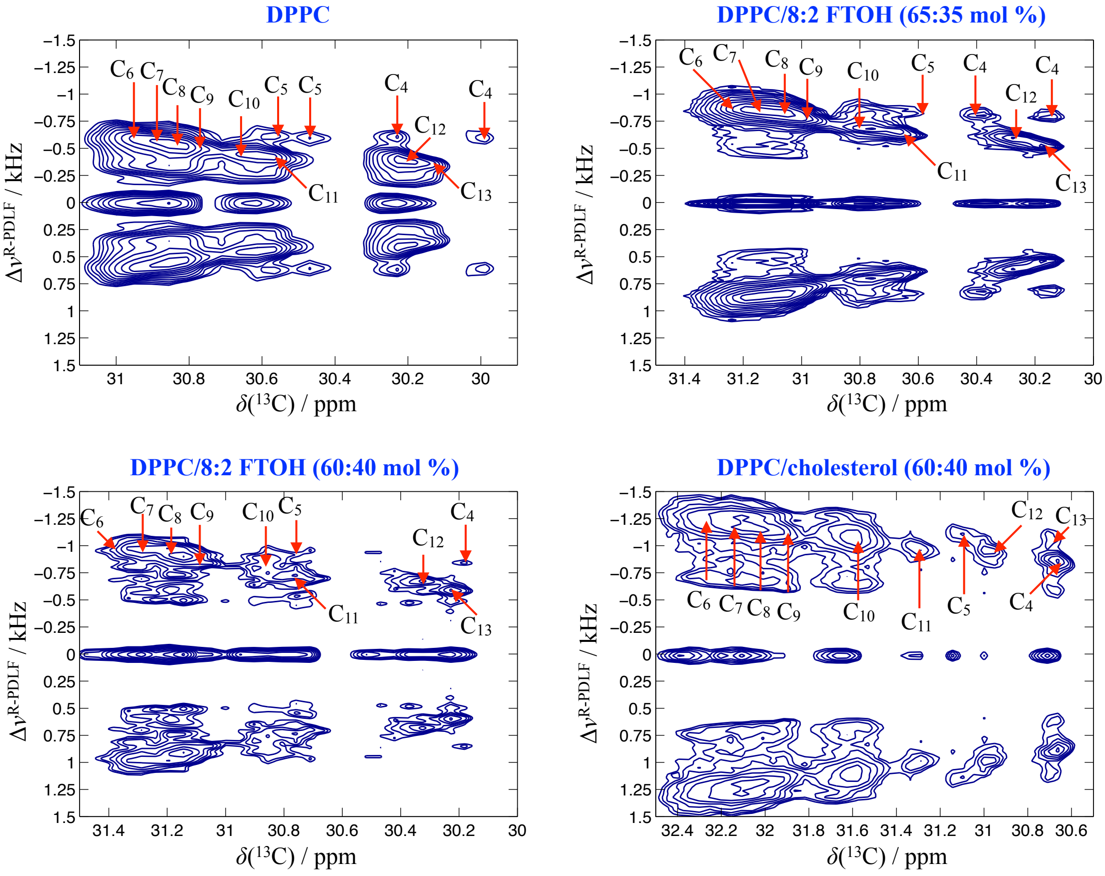

FIG. 3: R-PDLF spectral contour plots from the acyl chain crowded spectral region from 30 to 32.4 ppm for all the samples studied. The corresponding samples are indicated in the top of each plot. The dipolar slices selected from these contours for determining the C–H bond order parameter profiles (presented in Fig. 6 in the main text) are shown in the following figures. The assignment of the splittings were based on the previous assignment reported by Ferreira et al. for POPC MLVs (reference 36 in the main paper).

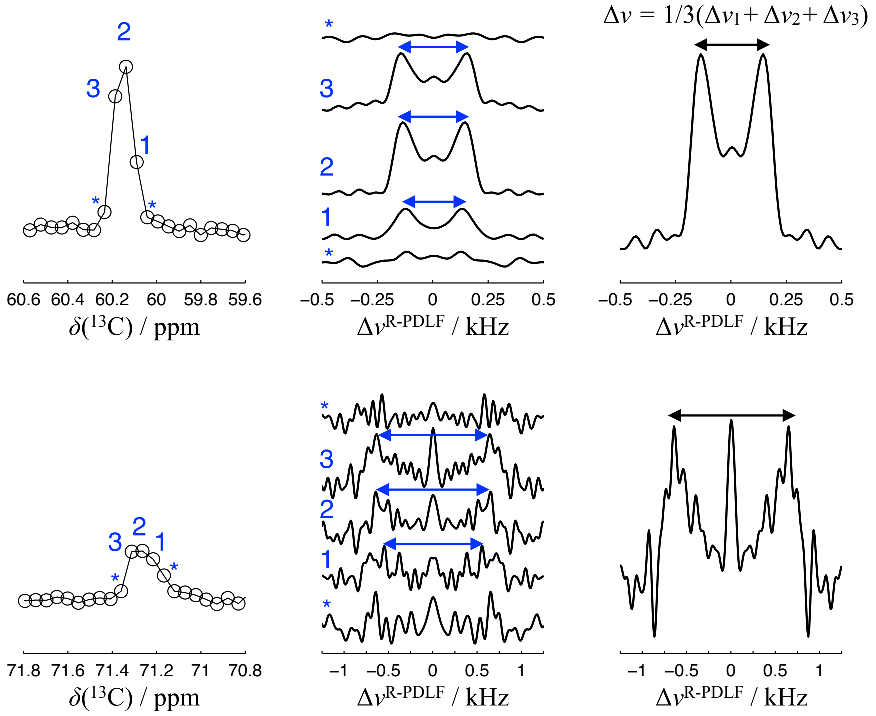

FIG. 4: Scheme to illustrate the calculation of the order parameter magnitudes, $|S_{\rm CH}|$ and error bars plotted in Figure 5 of the main manuscript. The upper and bottom plots show the R-PDLF spectrum of the α and g_2 carbons of DPPC, respectively, measured from DPPC/8:2 FTOH (60:40 mol %) MLVs. Due to both the higher signal-to-noise ratio and the smaller dipolar splitting of α, its R-PDLF splitting is highly resolved while for g_2 the location of the maxima in the dipolar slices is obscured by the low signal-to-noise ratio. In order to estimate the effect of noise in the determination of splittings from the dipolar slices we used the following procedure. For each peak assigned in the spectrum we have determined the three dipolar slices with highest intensities as exemplified in the plots on the right with labels 1,2 and 3 (the asterisks identify adjacent slices). For each of these dipolar slices we calculate a dipolar splitting by finding the position of the maxima in the dipolar slice as illustrated in the middle plots. Each order parameter reported in Figure 5 is then calculated by using the average splitting (over the three splittings determined) and Equation 5 in the main manuscript. The error bars are calculated by using the higher and the smaller of the three splittings. As illustrated in the plots on the right, each dipolar spectra shown in Figs. S6 to S9 are constructed by summing over the three slices used to calculate the order parameter for a given carbon while the arrow depicted shows the average splitting over the three slices.

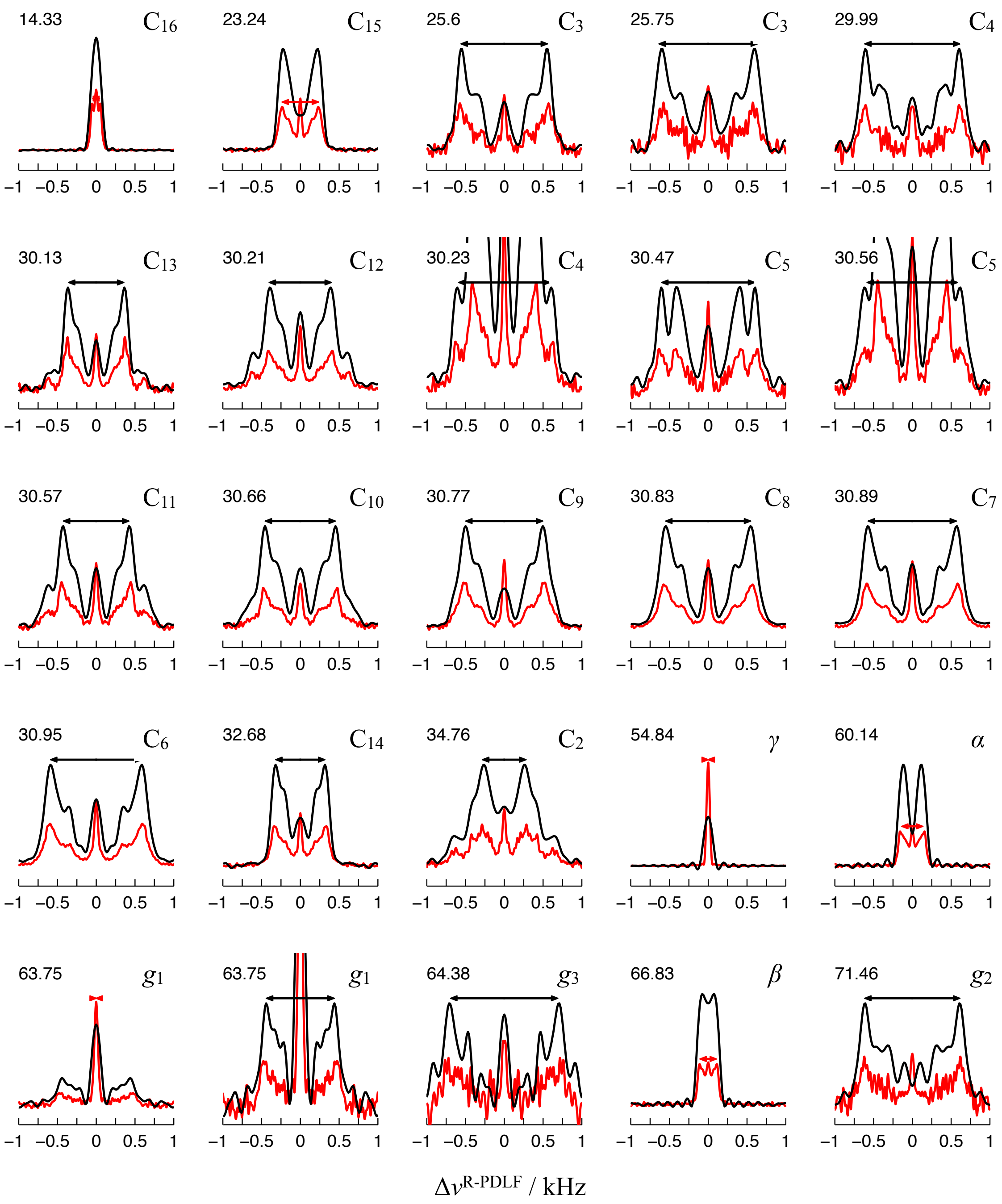

FIG. 5: R-PDLF dipolar slices acquired for DPPC MLVs. The labels on the right top corner indicate the carbons assigned to the ^{13}C chemical shifts selected indicated on the left top corner. The black dipolar slices refer to R-PDLF experiments using a total number of 512 scans for each point in the indirect dimension and a total number of 32 t_1 increments measured while red dipolar slices were acquired by using 128 scans and 64 t_1 increments. The use of a second experiment with higher number of t_1 increments was used to enable measurement of the smalest couplings.

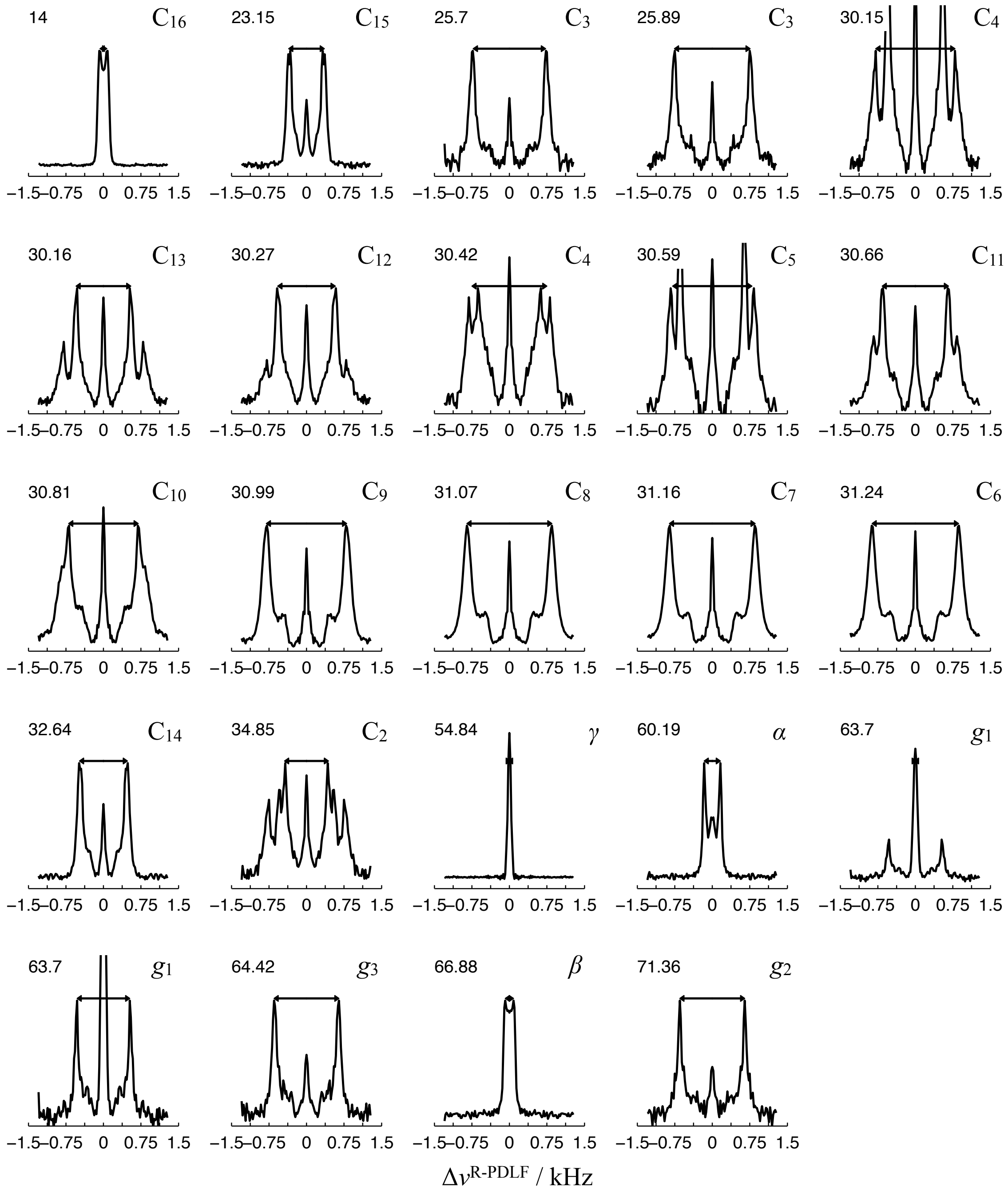

FIG. 6: R-PDLF dipolar slices acquired for the DPPC/8:2 FTOH (65:35 mol %) MLVs. The labels on the right top corner indicate the carbons assigned to the ^{13}C chemical shifts selected indicated on the left top corner. The dipolar slices refer to R-PDLF experiments using a total number of 512 scans for each point in the indirect dimension and a total number of 32 t_1 increments measured.

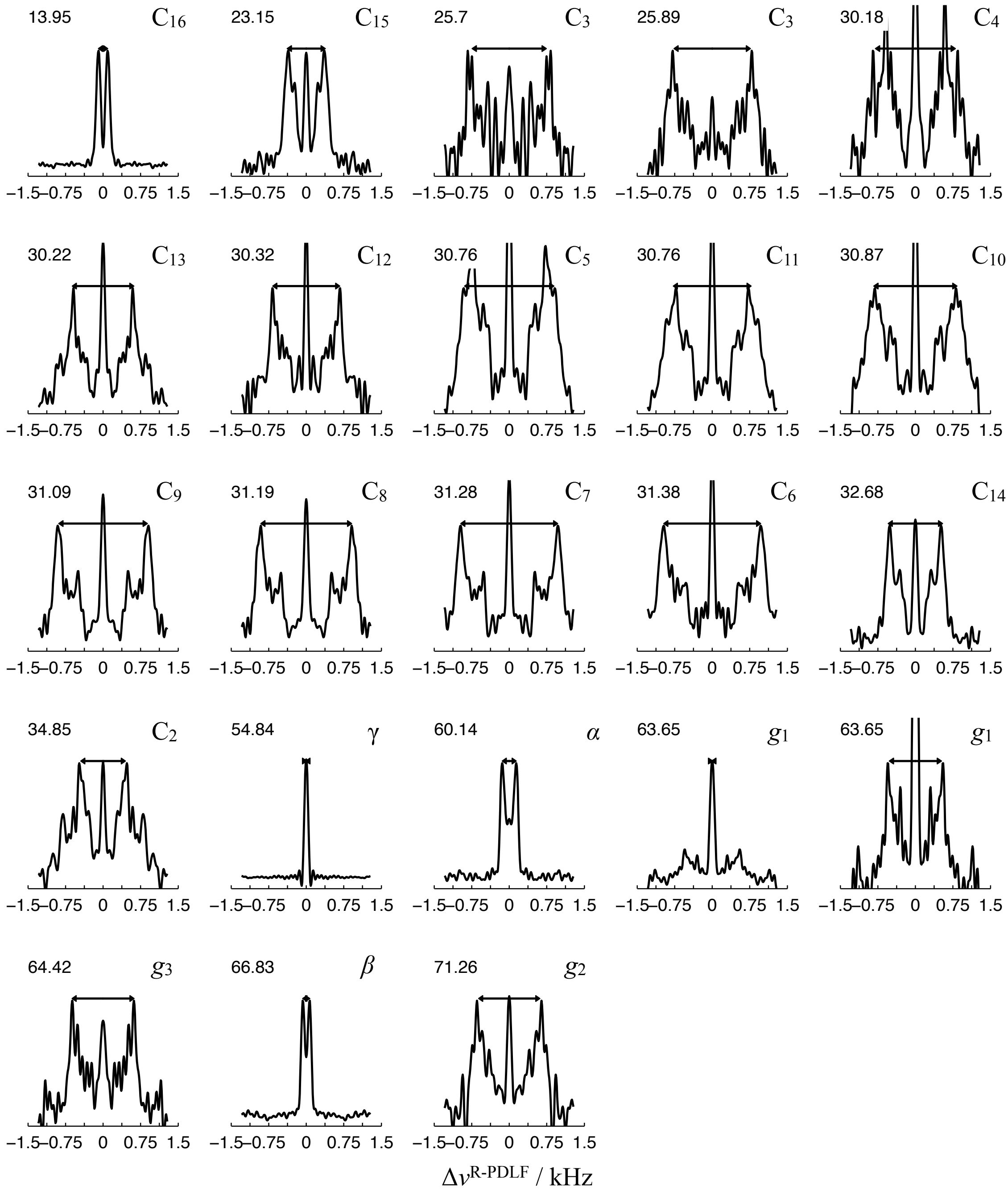

FIG. 7: R-PDLF dipolar slices acquired for the DPPC/8:2 FTOH (60:40 mol %) MLVs. The labels on the right top corner indicate the carbons assigned to the ^{13}C chemical shifts selected indicated on the left top corner. The dipolar slices refer to R-PDLF experiments using a total number of 512 scans for each point in the indirect dimension and a total number of 32 t_1 increments measured.

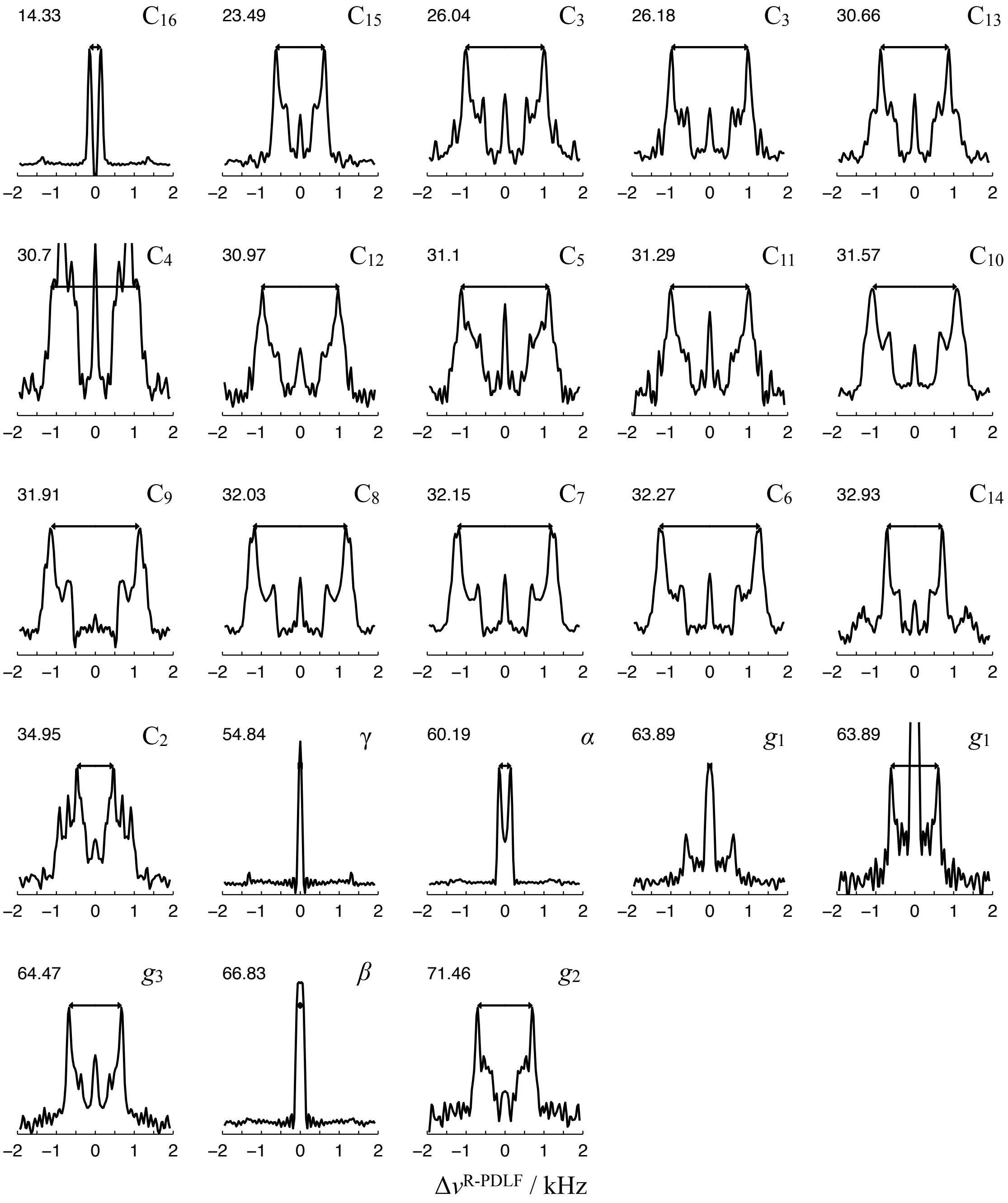

FIG. 8: R-PDLF dipolar slices acquired for the DPPC/cholesterol (60:40 mol %) MLVs. The labels on the right top corner indicate the carbons assigned to the ^{13}C chemical shifts selected indicated on the left top corner. The dipolar slices refer to R-PDLF experiments using a total number of 512 scans for each point in the indirect dimension and a total number of 32 t_1 increments measured.
